# Oral Drug Delivery Systems Comprising Altered Geometric Configurations for Controlled Drug Delivery

**DOI:** 10.3390/ijms13010018

**Published:** 2011-12-22

**Authors:** Kovanya Moodley, Viness Pillay, Yahya E. Choonara, Lisa C. du Toit, Valence M. K. Ndesendo, Pradeep Kumar, Shivaan Cooppan, Priya Bawa

**Affiliations:** Department of Pharmacy and Pharmacology, Faculty of Health Sciences, University of the Witwatersrand, 7 York Road, Parktown, Johannesburg 2193, South Africa; E-Mails: kovanya.moodley@students.wits.ac.za (K.M.); yahya.choonara@wits.ac.za (Y.E.C.); lisa.dutoit@wits.ac.za (L.C.T.); pradeep.kumar@students.wits.ac.za (P.K.); valence.ndesendo@wits.ac.za (V.M.K.N.); shivaan.cooppan@students.wits.ac.za (S.C.); priya.bawa@students.wits.ac.za (P.B.)

**Keywords:** controlled drug delivery, geometrically altered devices, multilayered tablets, polymeric materials, release modules assemblage

## Abstract

Recent pharmaceutical research has focused on controlled drug delivery having an advantage over conventional methods. Adequate controlled plasma drug levels, reduced side effects as well as improved patient compliance are some of the benefits that these systems may offer. Controlled delivery systems that can provide zero-order drug delivery have the potential for maximizing efficacy while minimizing dose frequency and toxicity. Thus, zero-order drug release is ideal in a large area of drug delivery which has therefore led to the development of various technologies with such drug release patterns. Systems such as multilayered tablets and other geometrically altered devices have been created to perform this function. One of the principles of multilayered tablets involves creating a constant surface area for release. Polymeric materials play an important role in the functioning of these systems. Technologies developed to date include among others: Geomatrix^®^ multilayered tablets, which utilizes specific polymers that may act as barriers to control drug release; Procise^®^, which has a core with an aperture that can be modified to achieve various types of drug release; core-in-cup tablets, where the core matrix is coated on one surface while the circumference forms a cup around it; donut-shaped devices, which possess a centrally-placed aperture hole and Dome Matrix^®^ as well as “release modules assemblage”, which can offer alternating drug release patterns. This review discusses the novel altered geometric system technologies that have been developed to provide controlled drug release, also focusing on polymers that have been employed in such developments.

## 1. Introduction

**Figure 1:**
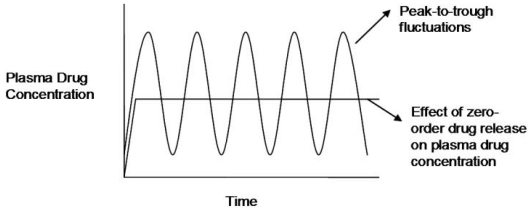
Plasma drug concentration *versus* time profile exhibiting the effect of zero-order drug release on plasma drug levels (adapted from Shahiwala *et al.* [15]).

Modified or controlled release oral drug delivery systems have, over the last few decades, been shown to offer advantages over conventional systems [1–6]. These include increased patient compliance [7,8], selective pharmacological action; reduced side-effect profile and reduced dosing frequency [9]. These systems may therefore have a significantly beneficial outcome in therapeutic efficacy. Controlled release offers prolonged delivery of drugs and maintenance of plasma levels within a therapeutic range [10,11]. Furthermore, by pairing drug administration rate with drug elimination rate, steady-state plasma levels can be maintained [12,13]. Currently most drug delivery systems exhibit first-order drug release kinetics where the plasma level of the drug is extremely high after administration and then decreases exponentially. This poses disadvantages such as minimal therapeutic efficacy due to reduced drug levels; or drug toxicity which can occur at high concentrations [14]. This type of drug release does not allow for appropriate plasma drug level balance. Peak-to-trough fluctuations (as depicted in Figure 1) may occur with first-order drug release that may cause dose dependent side effects [15,16].

Drug delivery systems should ideally exhibit zero-order drug release kinetics which allows for a constant quantity of drug to be released over an extended period of time, resulting in uniform and sustained drug delivery [2,17–21]. Zero-order is a desired drug release kinetic in antibiotic delivery, the treatment of hypertension, pain management, antidepressant delivery and numerous other conditions that require constant plasma drug levels [14,22,23]. Thus, various studies have been undertaken attempting to develop systems that are easily able to provide zero-order or near zero-order drug release [24–31].

The utilization of geometric principles have for many years been considered and employed in order to modify drug release behavior from non-linear to zero-order or near zero-order release kinetics [32–38]. Thus far researchers have attempted to control dissolution behavior of drug delivery systems by modifying and controlling the geometry of the employed devices e.g., geometries such as spherical, cylindrical, holed cylindrical and biconvex devices [39–41].

One of the principles involved in altering the geometry of tablets is to create a constant surface area for drug release to enable the achievement of zero-order kinetics [42,43]. Systems such as multilayered tablets, donut-shaped tablets, Procise^®^, Geomatrix^®^ and Smartrix^®^ technologies have been developed employing geometric manipulations [5,36,40,44,45]. These geometric manipulations may also be employed to develop drug delivery systems for the treatment of specialized biological conditions where zero-order drug release is not optimal, for example chronotherapy for heart conditions [46] or the scheduled treatment of asthma and inflammation [47]. Bimodal drug release may also be desirable with drugs that have variable absorption sites along the gastro-intestinal tract [12,48]. Technologies such as the Dome Matrix^®^ have shown promise in achieving varied drug release profiles in order to treat specific conditions [47]. Dilacor XR™ was developed as an extended release formulation for the delivery of diltiazem hydrochloride as described by Colombo and co-workers where the geometry of the system played an important role in the drug release profiles [49]. In addition, the polymeric materials used to construct these technologies play an important role in the functioning of these specialized systems [40,41]. Thus far, various types of polymers have been investigated for their ability to control drug release [50]. Polymers are the essential drug carriers of multilayered matrix tablets and their properties are an important factor in the behavior of these devices. In the past, polymers that were mainly employed for such purposes were the hydropolymers [40], while currently polymers investigated range from swollen and non-swollen [40,51], porous and non-porous [52–54] to erodible or non-erodible polymers [5,55].

In general, the mechanisms by which polymers perform their functions are by erosion [56], dissolution and swelling [57]. Some studies have shown that drug release from hydrophilic polymer matrices exhibit a typical time dependent profile in which the drug release is controlled ensuring swelling of the polymer [58–62]. This review thus discusses the application of altered geometric technology and its role in controlled oral drug delivery, focusing primarily on the types of polymers that have been employed in developing geometrically modified systems, the interplay of system geometry and polymeric selection ultimately contributing to the type of drug release patterns that are attained.

## 2. Multilayered Tablets for Controlled Drug Delivery

Mutilayered systems (bilayered, triple-layered, quadruple-layered, *etc*.) are becoming increasingly recognized as controlled-release drug delivery systems [63]. These systems have been shown to be advantageous over typical tablet systems as depicted in Table 1. Namdeo expressed that multilayered tablets have demonstrated promise, possessing various benefits, namely the ability to prevent interactions between drugs and excipients; and by providing an array of release profiles in one delivery system of either the same or different drugs, treatment for conditions that require a regimen of more than one drug, immediate drug release using a disintegrating monolithic matrix in order to achieve an initial peak in plasma drug level, delayed drug release using an eroding monolithic matrix which may deliver another active drug to a different part of the gastrointestinal tract, providing controlled drug release instituting a swellable monolithic matrix and better control and regulation of release profiles by retarding initial burst release and achieving zero-order kinetics [64]. It would be beneficial if research focused on further modification of these systems for improved and comprehensive drug release capabilities that enable a larger scope of application in drug delivery.

Controlled-release multilayered tablets typically involve a drug core layer that is surrounded by barrier layers that may be made up of hydrophilic swellable polymers such as hydroxypropylmethylcellulose (HPMC) and poly(ethylene oxide) (PEO) or hydrophobic polymers such as ethylcellulose (EC) [4]. The barrier layers minimize and therefore delay the interaction of the gastrointestinal environment with the active core, by decreasing the surface area available for drug release or by controlling the rate at which the solvent penetrates the layers [40]. This allows the initial burst release to be minimized and therefore the drug release can be controlled at a near constant level while the barrier layers undergo erosion or swelling [5]. The swollen barrier layers undergo erosion as time goes on, thus increasing the surface area which ultimately allows more drug to be released. Following the same principle, it is possible to obtain a constant release profile as well as other types of dissolution patterns such as pulsatile or delayed delivery as well as extended drug delivery depending on the characteristics of the polymers employed. In either case the system should ideally erode completely (*i.e.*, leaving no residue in the gastrointestinal tract after the entire amount of drug is released).

**Figure 2:**
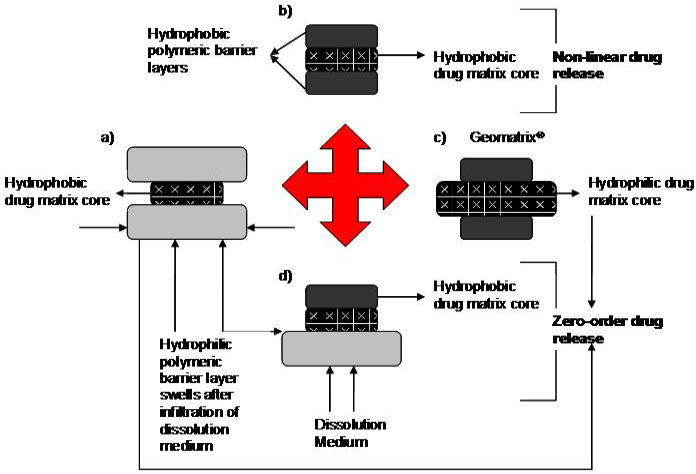
Various polymeric formulations of multilayered tablets and possible drug release behavior (adapted from Chidambaram *et al.* [65])

The different types of multilayered tablet designs with varying drug release behaviors are shown in Figure 2 [65]. There are multilayered tablets that can provide zero-order sustained release where the tablet consists of either a hydrophilic or hydrophobic core layer with barrier layers that are press coated to the surfaces of the core layer. This leaves the sides of the core layer exposed. It has been shown that generally constant drug release can be achieved when both barrier layers are hydrophilic and the core layer is hydrophobic [4,25]. However, other factors also need to be controlled in order to achieve zero-order drug release.

### 2.1. Geomatrix^®^ Multilayer Tablet Technology

**Figure 3:**
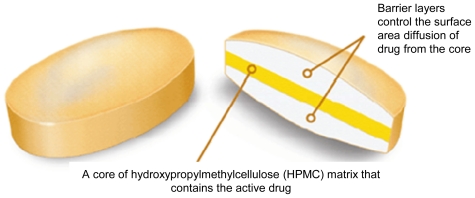
A typical Geomatrix^®^ multilayered tablet (Source: Shionogi Pharma, Inc. [67]).

The Geomatrix^®^ multilayer tablet technology was developed by Conte and co-workers for constant drug release [44]. The technology includes triple-layered and bilayered tablets. The triple-layered tablet which is exemplified in Figure 3 consists of an active core which is a hydrophilic matrix layer and two polymeric barrier layers on either side that are hydrophobic or semi permeable [66,67]. The bilayered tablet consists of the drug layer and one barrier layer [68]. The barrier layer modifies the swelling rate of the active core and reduces the surface area available for diffusion of drug [69–70]. Zero-order drug release can be achieved with the Geomatrix^®^ system [5]; however release is limited to one drug.

### 2.2. Sodas^®^ Multilayer Tablet Technology

**Figure 4:**
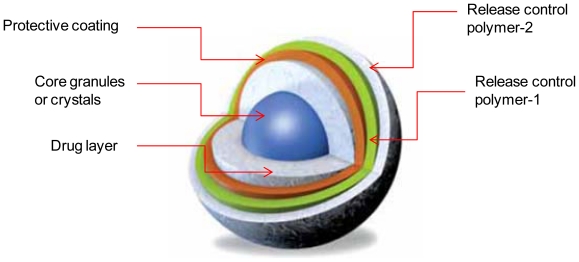
A schematic representation of Sodas^®^ multilayer tablet technology (adapted from Elan drug technologies [71]).

Sodas^®^ multilayer tablet technology (Figure 4) is a multilayer drug delivery system which focuses on the production of controlled release beads [71]. The Sodas^®^ technology is characterized by its inherent flexibility that enables the production of customized dosage forms that respond directly to individual needs such as pain and blood pressure. The technology essentially leads a pursatile drug release where the drug is released in pulses that are separated by defined time intervals. Examples of this technology include Ritalin^®^ LA and Focalin^®^ XR. They are both used to treat Attention Deficit Hyperativity Disorder (ADHD). They provide a once-daily pulsed profile that offers the patient efficacy throughout the day negating the need for taking the dose during working hours unlike the twice-daily dosing of the conventional immediate release tablet [71]. Benefits offered by the SODAS^®^ technology include: controlled absorption with resultant reduction in peak to trough ratios, targeted release of the drug to specific areas within the gastrointestinal tract, absorption independent of the feeding state, suitability for use with one or more active drug candidate, facility to produce combination dosage forms, “sprinkle dosing” by administrating the capsule contents with soft food, once or twice daily dose resembling multiple daily dose profiles [71].

The earlier described studies have provided practical technical ideas in the development of multilayered tablets depending on the clinical applications of these systems. The studies have also provided insight on what strategies need to be considered for further application. Table 2 provides the summary of the polymers influencing the behavior and release characteristics of multilayered tablets.

It is observed that there are great variations of multilayered tablet technology proving flexibility which affords possibilities for positive research development. With the intuitive selection of polymers and the appropriate employment of geometric principles, multilayered tablets may emerge as the future benchmark for the treatment of chronic diseases. However the difficulties that may occur with the scale up of more intricate layered drug delivery systems may be considered to be unfavorable to the pharmaceutical industry. The necessity of specialized equipment may add to the difficulties in commercialization of these systems.

### 2.3. Factors Affecting the Rate of Drug Release from Multilayered Tablets

#### 2.3.1. Polymers Employed in Multilayered Tablets

Generally, a multilayered system should initially swell, then gel and ultimately slowly erode [4,72]. A study done by Efentakis and co-workers investigated the effect of polymeric substances on drug release. Hydrophilic and swellable polymers such as HPMC (Methocel^®^ K100M), microcrystalline cellulose (MC) and PEO and the hydrophobic polymer cellulose acetate propionate (CAP) were employed in this study in which venlafaxine HCl was used as the model drug. The study focused on a core tablet that contained venlafaxine HCl and Methocel K100M as the drug carrier. Bilayered and triple-layered tablets were prepared using the core tablet. The bilayered tablet consisted of a core tablet where one surface was covered with either Cellulose Acetate Phthalate (CAP) or Methocel E50LV, while both surfaces of the core tablet were covered with both of the polymers to form the triple-layered tablets [40]. Hydrophilic polymers were employed as drug core matrices due to their swelling ability [73–76]. The release profiles obtained demonstrated that drug release was slower from the multilayered tablets than from the core tablet alone [40]. When the core tablet came into contact with the dissolution medium, it swelled and expanded. This caused an increase in the diffusion path length for the drug and the drug release rate was therefore reduced. Upon employing HPMC as a barrier layer, the layer swelled concurrently with the core tablet, merging the core surfaces thereby enveloping part of the core, which resulted in the limiting of drug transport through the barriers [40]. CAP did not swell due to its impermeability and therefore drug dissolution and the drug release rate was retarded. The use of HPMC or CAP in the barrier layers showed similar results in terms of retarding drug release except that Methocel showed slow erosion as opposed to CAP [40]. Generally, HPMC devices presented with slower drug release when compared to CAP devices, the reason being that they form a more efficient and solid barrier. Overall, the study showed that the characteristics of the polymers employed had a significant influence on the release profiles of the tablets although the choice of polymers employed in the study was conservative. Further research that focuses on the use of novel specialized polymers that are competent in providing zero-order drug release is necessary.

A study performed by Chidambaram and co-workers assessed the behavior of layered diffusional matrices for zero-order sustained drug release. Layered tablets were formulated with a hydrophobic core layer which contained the drug; this layer typically consisted of 24% ^w^/^w^ pseudoephedrine HCl, 40% ^w^/^w^ carnauba wax and lactose filler. The barrier layers were composed of either hydrophilic (Methocel^®^ K4M or K100M or Avicel PH 101) or hydrophobic polymers. Three different types of matrices were formulated. In the first type, the two barrier layers were hydrophilic, in the second type, one of the barriers was hydrophobic while the other was hydrophilic and in the third type, the two barrier layers were both hydrophobic [65]. Results showed that more desirable linear release profiles were obtained with the first and second type of matrices as depicted in Figure 2a,d, while the barrier layers in the third system needed to be manipulated in order to achieve zero-order release kinetics [25,65]. The proposed mechanism for the zero-order drug release from the first type of matrix was that as the hydrophilic barriers swelled and eroded, the rate of diffusion of drug from the hydrophobic middle layer decreased [65,77]. According to the study, the release rate from the lateral surface was influenced by polymer viscosity and concentration. These factors ultimately influence diffusion path length as well as the diffusion co-efficient. The use of polymers that possess mechanical or chemical characteristics to intrinsically alter the geometry, via modification of the diffusion path length, of matrices for controlled release may be an interesting perspective to study for future drug delivery research.

#### 2.3.2. Structure of the Device

A study undertaken by Efentakis and co-workers illustrated that the structure of a system plays an important role in its drug release behavior. They found that covering a larger area of the core tablet by a barrier layer results in the retardation of drug release to a greater extent, as it forms a more efficient barrier thereby decreasing the drug release rate [40]. Another study by Efentakis and Peponaki re-iterated the significance of structure and geometry of triple-layered tablets with isosorbide mononitrate as a model drug. The weight and thickness of the barrier layers also had a pivotal role in drug release behavior [70]. Chidambaram and co-workers established that drug release from the surfaces of the core was dependent on the thickness of the hydrophilic barrier layers [65]. An investigation by Streubel and co-workers looked at bimodal drug release from multilayered matrix tablets. It was discovered that by increasing the weight of the barrier layers from 50 mg to 150 mg it resulted in a more effective retardation of drug release, thus it was concluded that by manipulating the weight and thickness of the outer layers (as shown in Table 2) a desirable drug release profile of individual drugs may be achieved, thus complementing their pharmacokinetic behavior [69]. The concept of barrier layers have proven to be beneficial in multilayered tablet designs; however converting the barrier layers into additional controlled release drug matrices may hold further potential for future application [64].

**Figure 5:**
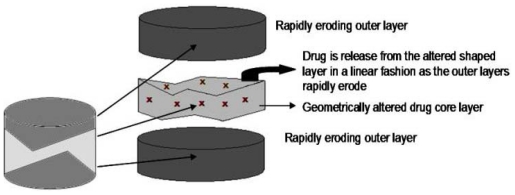
Smartrix^®^ technology (Adapted from Zerbe and Krumme [78]).

Zerbe and co-workers have shown that there are also complex multilayered tablet systems with layers of various shapes that are able to provide zero-order drug release. The Smartrix^®^ tablet technology (Figure 5) that was developed by LTS Lohmann Therapie-Systeme employs modified geometrical shapes that compensate for the varying surface area caused by erosion or swelling (displayed in Table 3) [78]. The triple layered tablet is composed of a drug core that has a specific shape. The core is enclosed between two rapidly erodible outer layers. The middle layer has a biconcave shape that the two outer layers tightly bond to after compression. The thickness of the outer layers and the shape of the drug core control the release of drug usually in a linear fashion. The Smartrix^®^ system is also able to achieve bimodal drug release [79], as an added advantage of being flexible. This technology has proven to be useful as it does not require specialized polymers to perform the desired function. The study that emanated in the development of the Smartrix^®^ system has further emphasized the functionality of shape and geometry in altering drug release behavior. However, this technology requires specialized dry tablet press machines that may pose as a disadvantage.

### 2.4. Bilayered Tablets

Bilayered tablets have proven to be effective in delivering drugs that require a loading dose followed by a maintenance dose [80–83]. Commonly, in bilayered systems, one layer contains a quantity of drug for conferring immediate release, while the second layer contains a quantity of drug for extended release. The rapid release layer disintegrates immediately after administration while the matrix layer remains intact during the passage of drug through the gastrointestinal tract. The matrix erodes in a controlled fashion in order to maintain blood levels. Two drugs may also be incorporated into this delivery system for variable release profiles. A bilayered tablet for the delivery of propranolol hydrochloride was developed by Patra and co-workers. These tablets were comprised of an immediate release layer and a sustained release layer. Sodium starch glycolate was employed as the superdisintegrant in the rapid release layers of various formulations, while the polymers Eudragit^®^ RL, Eudragit^®^ RS and EC were utilized in the sustained release layers. Drug release studies illustrated that there was an initial burst release that delivered the loading dose while the rest of the drug was released over 12 hours in a sustained manner [81]. The same concept has been demonstrated in a patent by Kim and co-workers where the system provided release of two drugs in different manners. The controlled release layer delivered metformin while the rapid release layer delivered glimepiride. The controlled release layer was made up of a mixture of hydrophobic and hydrophilic polymers, while the immediate release layer was composed of a disintegrant and glimepiride [82]. This further emphasizes the positive function of these systems in treating chronic conditions such as hypertension and diabetes. Nirmal and co-workers developed a bilayered tablet containing atorvastatin calcium for immediate release and nicotinic acid for extended release for the concurrent treatment of hypercholesterolemia. It has been shown that the combination of these two drugs results in an important reduction of low density lipoprotein cholesterol as well as desirable variations in high density lipoprotein cholesterol [83]. Methocel^®^ K100M was employed as the polymeric matrix for nicotinic acid and the immediate release layer containing atorvastatin calcium was formulated using super disintegrant, croscarmellose sodium. Drug release studies were performed over 12 hours and the results indicated that these tablets were successful in delivering two types of drugs concurrently [83]. This bilayered system design may thus be valuable for future application in the successful treatment of hypertension.

VersaTab^®^ Bilayered Tablet Technology

**Figure 6:**
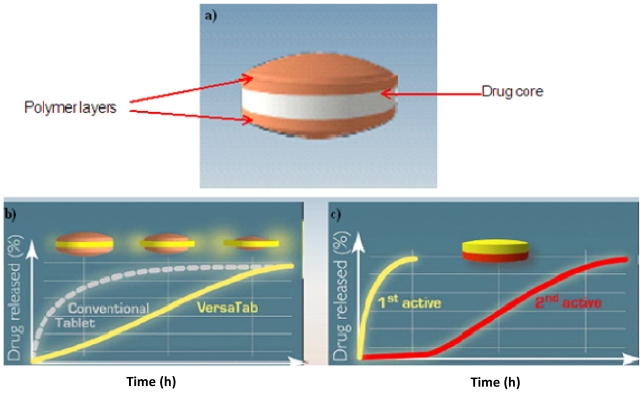
(**a**) VersaTab^®^ bilayered tablet; Profiles depicting VersaTab^®^ bilayered tablet technology: (**b**) One bioactive-controlled release; (**c**) Two bioactives-immediate release and controlled release.

A VersaTab^®^ bilayered tablet technology is a tablet that has been devised to result in a linear drug release through controlled erosion [84]. The technology designs tablets with the ability to co-release multiple drugs with different release rates. This technology is suitable for a large number of bioactives. The tablet is highly versatile with a broad range of delivery profiles and therefore endowed with improved patient compliance. Figure 6 displays profiles that depict VersaTab^®^ bilayered tablet technology firstly with one bioactive that provide controlled release and secondly with two bioactives that provide immediate release and controlled release [84].

### 2.5. Triple-Layered Tablets

Triple-layered tablets are comprised of an inner drug core layer which is sandwiched between two surrounding barrier layers [4,64]. These barrier layers may also contain drug and serve as matrices to release drug in various release patterns [64]. The general mechanisms of action of triple-layered tablets include erosion of matrix layers, creation of a drug concentration gradient, limiting surface area of release of the swellable matrix by the barrier layers, erosion and swelling of the barrier layers to achieve a constant area for uniform drug release, as well as varying of the layers dissolution to achieve pulsatile or alternating release profiles [40,64]. Triple-layered systems have some rewards in contrast to typical systems due to the varying release pattern capability, simplicity of manufacturing, reduced dosing frequency that leads to enhanced patient compliance, enhanced safety profile of drug levels and reduced cost.

#### 2.5.1. Geolock^TM^ Technology

**Figure 7:**
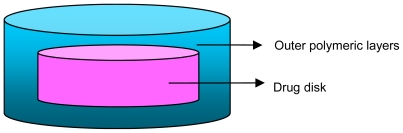
A schematic of a triple layered Geolock^TM^ tablet (Adapted from : SkypePharma [85]).

Geolock^TM^ Technology is a triple layered tablet that has been devised for chronotherapy focused-real time oral drug delivery [85]. Principally, it is a new clinically oral drug delivery technology which allows, with a high degree of precision, the timed delivery of drugs that employs a press-coating technique. Geolock^TM^ tablet is composed of an active drug core (middle layer) that is surrounded by two outer protective layers (Figure 7). The inner core can be a single or combination of drugs essentially formulated for either immediate or modified release.

#### 2.5.2. Various Drug Release Profiles Achievable by Triple-Layered Tablets

It has been shown that both immediate and sustained drug delivery can be obtained through a single triple-layered tablet [4]. In this case, there is an initial immediate rapid release of drug, which is followed by a sustained constant drug release. This type of release could be useful for drugs that need a high plasma concentration immediately for therapeutic efficacy where zero-order drug release kinetics is not required. Maggi and co-workers developed such a system where a quick/slow release of Naproxen was achieved [86]. A multi-layered controlled release tablet containing naproxen and naproxen sodium salt was developed by Desai in 1996. The tablet composition included a layer containing naproxen which offered a delayed release of a granulated form of naproxen and another immediate release layer that contained naproxen sodium salt. This system was designed to deliver a prompt therapeutic effect while maintaining the effect for 24 hours [87]. This type of variable release is extremely useful for the delivery of specific drugs that need both rapid and sustained release.

A patent by Iyer and his co-workers reported on a triple-layered system that was comprised of a sustained release layer containing methylcobalamin while the other two immediate release layers each contained an antihypertensive, and a lipid regulator or a serum homocysteine lowering agent providing a valuable combination for the treatment of hypertension [88]. Compressed mini-tablets were designed for biphasic delivery of drugs with zero-order release kinetics [88,89]. The mini-tablets were compressed within an outer filling that was composed of MC (Avicel PH102) that filled the space between the minitablets, this outer filling provided rapid drug release while the minitablets provided prolonged release [88]. The mini-tablets specifically were composed of either HPMC or EC, with a diameter of 2.5 mm and an approximate weight of 12 mg each. Various formulations that differed in the amount of outer filling and number of mini-tablets used were prepared. Results showed that the mini-tablets with HPMC showed the most potential for achieving zero-order drug release. Another patent by Zerbe and co-workers presented a multilayer triple-layered oral system that had a matrix core that contained an NSAID for sustained release and two surrounding layers each containing an H_2_-receptor antagonist. The first layer provided sustained release of the antagonist and the second layer provided a rapid release of the antagonist. This idea was developed for use in the treatment of osteoarthritis in people who are more susceptible to developing gastrointestinal adverse effects such as NSAID-induced ulcers [63].

Time-programmed or chronotherapeutic drug delivery can also be achieved with multi-layered tablets [4]. With chronotherapy, drug release is governed by time so that drug is released only when needed in accordance with the circadian rhythms of the body. This can be described as pulsatile release rather than continuous release. This type of delivery may be beneficial for preventing tolerance arising from the drug as well as in reducing the side-effects. Press coating is a good technique for producing this type of time-dependent release. With press coating, there are no specific coating solvents or equipment that is needed and therefore the manufacturing process is more efficient [90]. The core layer is essentially coated with polymeric barrier layers by compression. The drug is released when the barrier layers either swell or erode. The coating delays the interaction of the core with the fluid medium, which causes a lag time before the drug is released. When the solvent penetrates the core layer, the core swells and dissolves, this causes the coating shell to break thereby rapidly releasing the drug [4]. This research further confirms the useful flexibility of triple-layered tablets although the application is often limited to the release of one drug.

It has been demonstrated that bimodal drug release may also be necessary when a non-uniform drug release rate is desired [69]. The mechanism of release typically involves an initial rapid release, followed by a slower constant release, which is then followed by another rapid release period. Bimodal drug release may be advantageous in that the initial rapid release phase which is followed by a slow release phase is able to compensate for the slow absorption of drug from the stomach and small intestine. More uniform delivery of drug into the systemic circulation can be achieved because bimodal release systems increase the rate of drug release when the ability of the body to absorb the drug decreases. Streubel and co-workers developed multilayered matrix tablets that could achieve bimodal drug release rates. Hydroxypropyl methylcellulose acetate succinate (HPMCAS) was used to form the matrix due to the fact that its solubility varies with pH. It is in essence, water soluble at high pH values and water-insoluble at low pH values. The study aimed at determining the effect of HPMCAS on drug release from the different layers of the tablet [69].

The various geometries of triple-layered tablets may be quite useful for controlling the delivery of highly water soluble drugs. A study by Siahi and co-workers investigated the development of triple-layered tablets for the delivery of verapamil hydrochloride in a controlled manner. The tablets consisted of three layers that were prepared by compressing polymers which were either natural or semi-synthetic onto the sides of the drug core. HPMC, acacia and tragacanth were used as drug release delaying layers encompassing the core. Different formulations containing separate and combined amounts of these polymers were prepared. The results indicated that when tragacanth was used as a carrier, the release was delayed to a greater extent than when acacia was used. Results also showed that the location of the polymers in the triple-layered tablets had a substantial effect on the release kinetics [91].

Triple-layer guar gum matrix tablet formulations were developed by Krishnaiah and co-workers in which the controlled delivery of water-soluble drugs using guar gum as a carrier was explored. The system was evaluated in terms of the release rate of trimetazidine dihydrochloride from the matrix. Different concentrations of guar (30% ^w^/^w^, 40% ^w^/^w^ and 50% ^w^/^w^) were used to prepare the triple-layered tablets. The guar gum acted as a release retardant. The release rate from these formulations enabled a twice daily administration of the delivery system [92].

## 3. Multilayered Osmotic Devices

An indented core tablet strategy for preparing monolithic osmotic pumps was developed by Longxiao and co-workers. The tablet was compressed by a punch using a needle. The indented core was coated using EC as a semipermeable membrane coating and polyethylene glycol (PEG) as a plasticizer together with sodium chloride as an osmotic agent, controlled membrane permeability. The tablet was developed for the delivery of atenolol and sodium chloride and was used as an osmotic agent. Results showed that the tablet was capable of delivering the drug constantly over 24 hours and was not dependent on the agitation or release medium [93]. This system does not require specialized laser drills to form the orifices, thus reducing manufacturing costs. The study was useful in attempting to develop a system that functioned as efficiently as an osmotic pump, however, in a more feasible and cost-effective manner.

Longxiao and co-workers also prepared a bilayer osmotic pump tablet using the indented core strategy. The model drug used in the study was nifedipine. A modified tablet punch was used to prepare the tablets whereby the punch formed an indentation in the centre of the drug surface layer. The indentation was sprayed with a coating solution with only the bottom of the indentation being sufficiently coated. The sides of the indentation were not completely coated, which left an aperture where drug release could occur. The tablet demonstrated success in delivering nifedipine at a relatively constant rate for 24 hours [94].

**Figure 8:**
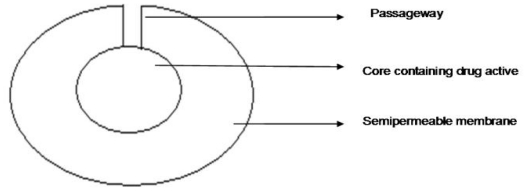
Schematic depiction of a multilayered osmotic device (Adapted from Fanner *et al.* [96]).

A US patent by Faour and co-workers described a multilayered osmotic device that could deliver more than one pharmaceutical agent. The device was developed to deliver the first therapeutic agent by immediate release and the second in a controlled manner. The device consisted of a core that contained a therapeutic agent, an osmotic agent and poly (vinylpyrrolidone) (PVP). A similar device patented by Fanner and co-workers consisted of a core was surrounded by a semipermeable membrane (Figure 8) that consisted of cellulose acetate esters and poly(ethylene glycol) (PEG) and contained a preformed passageway [95]. The semipermeable membrane was essentially permeable to the dissolution environment and impermeable to the therapeutic agent in the core. The device was also coated with a poly(vinylpyrrolidone)-(vinyl acetate) copolymer that partially or completely surrounded the semipermeable membrane and covered the passageway in the same manner as a plug. The final segment consisted of an external coat that was comprised of PVP and PEG and a second therapeutic agent that would be immediately released. When the external coat released the second agent, it eroded or dissolved thereby releasing the therapeutic agent contained in the core in a controlled manner.

## 4. Multilayered Floatable Drug Delivery Systems

A study that was conducted by Fassihi and co-workers investigated zero-order release kinetics from a self-correcting floatable asymmetric configuration drug delivery system. Theophylline was the model drug while PEO polymers of various molecular weights were employed as drug carriers. The various types of PEO polymers, drug and excipients were directly compressed into a triple-layer asymmetric floatable device. The core layer contained theophylline while the outer layers were composed of polymers and excipients in order to delay the interaction of the core layer with water, thereby delaying and controlling the drug release. Dissolution studies over 16 hours showed that the entire amount of drug was released in a zero-order manner with no initial burst release [9]. The release was dependent on the thickness of the layers and polymers used.

Fassihi and co-workers also investigated zero-order delivery of alfuzolin hydrochloride via a gastroretentive system. Triple-layered and bilayered matrices were developed by compressing PEO, HPMC, sodium bicarbonate, citric acid and PVP. Dissolution studies demonstrated the ability of the matrices to achieve floatation in pH 2 and pH 6.8 as well as providing zero-order drug release [97]. This system showed fine potential for providing enhanced bioavailability and targeted delivery to the small intestine.

A study by Yang and co-workers proposed a drug delivery system that would be able to treat *Helicobacter pylori*-associated gastric ulcers. The system was comprised of a swellable, asymmetric triple-layered tablet that was also floatable so as to increase the gastric retention time of the system. The employed rate-controlling polymers were HPMC and PEO. The core layer of the triple-layered tablet contained the drugs tetracycline and metronidazole. *In vitro* studies exhibited a sustained delivery of the two drugs over 6–8 hours, while the tablet was retained, showing the potential to achieve localized treatment, thereby improving therapeutic efficacy. This study poses great potential for the essential eradication of *Helicobacter pylori* infection that often results in hospitalization of patients who develop serious ulcers [98].

A patent awarded to Doshi and co-workers described a floatable drug delivery system that is able to deliver multiple drugs. The system was bilayered and was able to deliver a drug from one layer immediately, followed by slow and controlled release of another drug from the other matrix-forming layer. The immediate release layer contained a disintegrating agent while the matrix-forming layer consisted of a gas generating component and a gelling agent [80]. The aim of this system was to attain a controlled delivery of fluoroquinolones and to maintain the plasma levels of the drugs within a therapeutic range with once daily administration.

## 5. Core-in-Cup Devices

Danckwerts developed a core-in-cup tablet system that was able to provide zero-order drug release of aqueous-soluble and aqueous-insoluble drugs. The system consisted of a disc-shaped matrix core that was compression-coated on one surface as well as at the circumference in order to form a cup around the core. Drug was released in a sustained manner from one stable surface that had a constant surface area. By manipulating the grade, quantity and exposed surface area of any hydrophilic polymer or mixture of polymers that erode constantly over time, the core-in-cup compressed tablet is able to deliver a constant amount of drug over time [99]. Results showed that the system was able to provide zero-order drug release for time intervals between 8 and 23 hours, the time of linear release was approximately 8 hours when 5% ^w^/^w^ HPMC K4M with caffeine core-in-cup tablets were produced and approximately 23 hours when 15% ^w^/^w^ HPMC K15M in ibuprofen core-in-cup tablets were produced. The research that has been conducted on core-in-cup devices showed several interesting and useful techniques as well as beneficial application in terms of the solubility of drugs, the flexibility of delivering both aqueous soluble and aqueous insoluble drugs pose an advantage.

**Figure 9:**
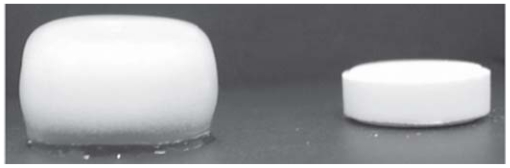
Typical geometries of core-in-cup tablets (Source: Guimarães *et al* [100]).

Danckwerts also studied the effectiveness of cup tablets of different depths for use in core-in-cup tablets and the optimal formulation in terms of drug release behavior. He developed a specific punch that is able to change the depth of the cup tablet, thus allowing it to carry various cores in terms of hardness and mass. The efficiency of cup tablets with varying depths and the optimal formulation in terms of drug release were investigated in the study. The cup tablets were composed of 15% ^w^/^w^ carnauba wax in EC while the core tablets were composed of 5% ^w^/^w^ HPMC K4M in ibuprofen. The results indicated that Ibuprofen was released at a near zero-order rate for 18 hours for the cup tablets that had a final depth of 4 mm [99]. Figure 9 shows the typical geometries of core-in-cup tablets.

## 6. Procise^®^ Technology

**Figure 10:**
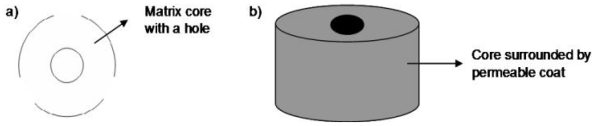
(**a**) Aerial schematic of Procise^®^ technology; (**b**) Two-dimensional schematic of Procise^®^ technology (adapted from Porter [79]).

The Procise^®^ device has a specific geometric configuration (as depicted in Figure 5) that controls drug release behavior [36]. It is composed of a core which contains uniformly dispersed drug with a core hole in the middle (Figure 10). It has been made known that, altering the geometry of the core can change the drug release kinetics into zero-order or even first order if desired (as indicated in Table 2). The core’s entire surface besides the surface of the cylindrical face is surrounded by a permeable inactive coat so that drug release occurs solely from the cylindrical area. The device is also able to deliver up to two drugs simultaneously with varying release profiles [36]. This technology further adds to the varied geometrical systems for flexible and simplified drug delivery.

## 7. Donut-Shaped Devices for Controlled Drug Delivery

There are various publications dating rather far back that established that zero-order release kinetics could be achieved from a hemispherical device containing a hole [42,45,101–105]. A study by Kim investigated drug release from uncoated compressed tablets that contained a single central hole. The impact of the hole size and drug solubility on drug release was also investigated. The tablets were composed of PEO and had a diameter of 12 mm. Theophylline was used as the model drug. The tablets provided zero-order drug release for approximately 80–90% of the drug followed by a decreased drug release rate. The results also indicated that as the size of the hole increased, the rate of drug release also increased. Drug solubility proved to be inversely proportional to duration of linear drug release [104]. It was concluded that the donut-shaped PEO tablets with an aperture were capable of providing zero-order drug release as the effect of surface area on release kinetics is reduced. The geometric factors that influence the drug delivery of donut-shaped tablets are shown in Table 2.

**Figure 11:**
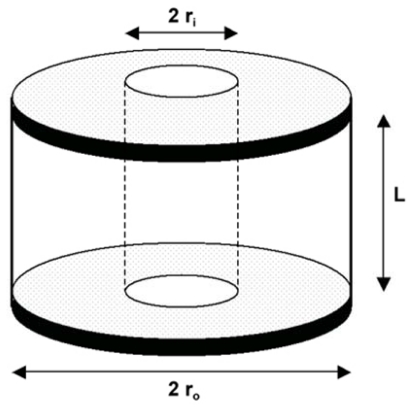
A schematic of a triple-layered, donut-shaped tablet (adapted from Kim *et al.* [66]).

The hydrophilic polymer based donut-shaped tablets developed by Kim, exhibited a disadvantage as they adhered to biological tissues and solids causing dose dumping of the drug [45]. It was due to this reason that Kim, decided to undertake a study on coated donut-shaped tablets for parabolic and linear drug release. Two types of polymers, namely rapidly erodible and slowly erodible polymers, were investigated. Zero-order release was achieved when the slowly erodible polymers were used and parabolic drug release was achieved when rapidly erodible polymers were used when diltiazem hydrochloride was incorporated as a model drug. It was also found that the drug release characteristics depended on the stirring rate and hole size [45]. These tablets did not adhere to either biological tissues or solids, thus proving to be more effective than the uncoated systems [45]. HPMC donut-shaped tablets were developed and investigated by Cheng and co-workers. Theophylline and diltiazem hydrochloride were employed as model drugs. Results depicted that zero-order kinetics was achieved for approximately 90% of the duration of the study. An increase in the size of the centre hole caused an increase in the rate of drug release and a longer duration of zero-order release [105]. A further study was conducted by Kim on triple-layered donut-shaped tablets (Figure 11) with enteric polymers to evaluate their controlled release ability. The tablets were prepared by layering three powders and compressing them with a punch. HPMCAS was the fundamental polymer of the core while the outer layers were composed of EC. The results showed that the solubility of the drugs had an effect on release kinetics. The hydrochloride salts of weakly basic drugs had a slower release rate than neutral drugs [66]. Thus, this system is capable of providing zero-order drug release for drugs with varying solubilities.

A study completed by Sundy and co-workers developed a novel compression-coated doughnut-shaped tablet for zero-order sustained release. The tablets were also assessed for their reproducibility. The tablets were prepared using a unique designed punch set. Hydrophilic and hydrophobic polymers were employed and evaluated as the coating layers. Caffeine and ibuprofen were used as model drugs as they have different solubilities and allowed for a comparison on the drug release profiles. Results showed that a coating layer of HPMC K15M and a core layer of HPMC K4M provided zero-order release of both caffeine and ibuprofen [42]. The tablets also proved to be feasible to manufacture on a larger scale.

## 8. Dome Matrix^®^ and “Release Modules Assemblage” Technology

**Figure 12:**
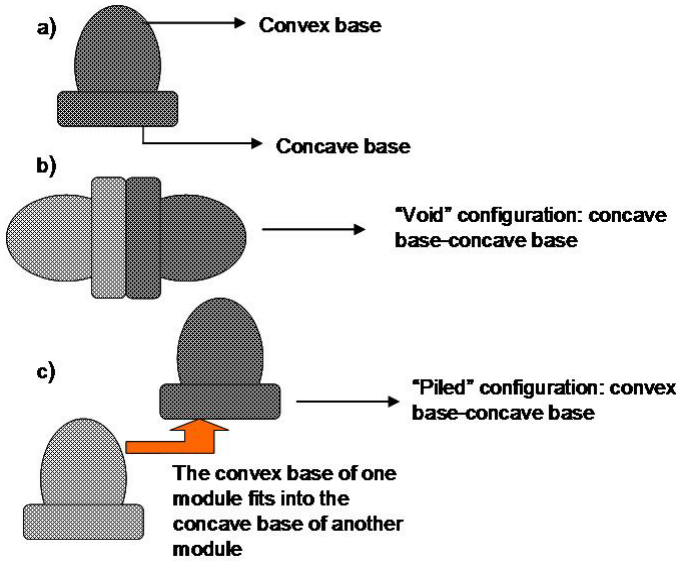
(**a**) Dome matrix^®^ module; (**b**) “void” configuration; (**c**) “piled” configuration (Adapted from Losi *et al.* [47]).

The dome matrix technology (described in Table 3) was developed by Losi and co-workers. The elementary module (depicted in Figure 12a) is a swellable matrix device comprising of a concave base on one end and a convex base on the other end. Losi and co-workers also developed the “release modules assemblage” technology which implies the creation of different drug delivery systems, having various functions by the assemblage of two or more of the elementary/release modules. Figure 6 illustrates the dome matrix modules and examples of the possible assemblages that may be achieved. The drug release patterns from these assemblages depended on the manner in which the modules were placed or attached to each other [47]. For example, multi-kinetics can be achieved and the delivery of two drugs in a single unit at a specific time and at a specific rate can is also possible. Two types of assemblages were mentioned in this study, the first was called “piled configuration” (depicted in Figure 12c) where the convex base of one module is inserted into the concave base of another module and the second was called “void configuration” (depicted in Figure 12b) where the concave base of one module is fixed onto the concave base of another module creating a space/void between the two modules [47].

The modules were compared to flat base matrices in terms of drug release behavior. Buflomedyl pyridoxal phosphate (BPP) was the model drug used in the study. The results depicted that the modules did not completely alter the kinetics compared to flat base matrices having the same weight and composition. However, the dome matrix^®^ had a higher initial release rate. The concave and convex bases themselves exhibited different release patterns, with the convex base releasing drug faster [47]. The technology proved to have potential benefits in terms of providing flexible drug release by increasing the amount of modules in an arrangement. This technology has various advantages although the complexity of the systems may be a shortcoming of the system in terms of administration of the devices to patients. A summary of various technologies that utilize geometric factors in drug delivery is provided in Table 3.

## 9. Conclusions

It has been elucidated that geometrically altered drug delivery systems, especially multilayered tablets, have provided various advantages to drug delivery technology. The ease of manufacture of these systems adds further benefit in terms of cost. These systems therefore show promise for therapeutic use in the future. The technology that these systems encapsulate, offers valuable knowledge and insight for the inspiration of more intricate and constructive drug delivery systems for wider applications. Future research may thus focus further on modifying these systems and using the basic technological principles to develop novel systems that may be able to be applied in broader and more complicated drug delivery, such as in the treatment of more complex diseases with a larger drug regimen that requires more individualized types of drug release. With the appropriate selection of polymer matrices and specialized geometries, these systems could be used to deliver more drugs in a more controlled manner for adequate time periods. The fact that drug delivery systems with altered geometric configurations (particularly tablets) have shown promising results in drug delivery technology and ease of manufacturing is an added advantage to the pharmaceutical industry.

## Figures and Tables

**Table 1:** Advantages of multi-layered tablets over conventional tablets (Adapted from Namdeo [64]).

Conventional Tablet	Multi-Layered Matrix Tablets
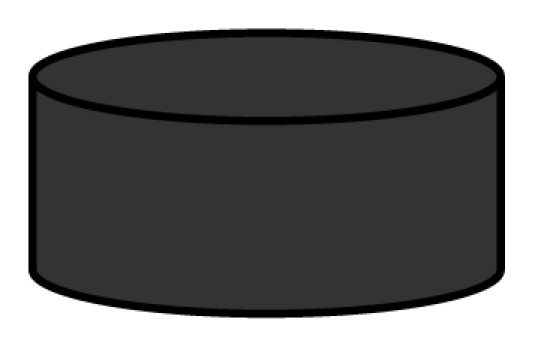	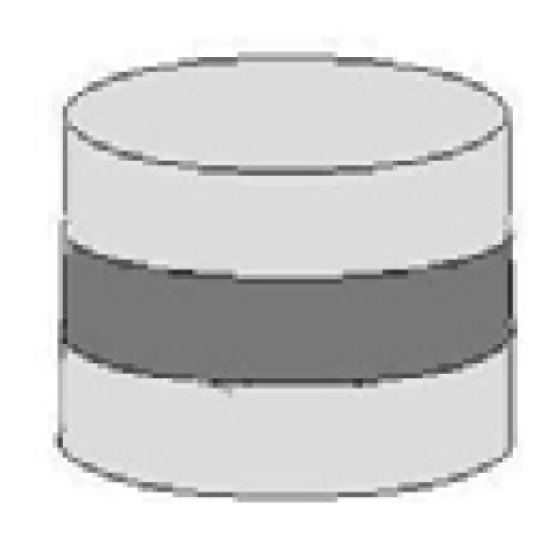
Drug is released in only one kinetic model	May be used to incorporate more than one drug and separate them if any chemical incompatibilities exist.
If more than one drug is incorporated, there is no way of avoiding chemical incompatibilities.	Drug release behavior is not restricted to one type, this system may offer varied drug release kinetics of the same or different drugs such as extended and immediate release.

**Table 2:** Summary of the type of polymers influencing the behavior and release characteristics of multilayered tablets.

Type of Polymer Used as Drug Carrier	Type of Polymer Used in Barrier Layers	Type/Dimensions of Tablet	Drug Release Achieved
Hydrophilic	Hydrophilic	Bilayered tablet	Extended drug release
Hydrophilic	Hydrophobic	Bilayered tablet	Drug release retarded to lesser extent
Hydrophobic	Hydrophilic (Methocel^®^ K4M)	Triple-layered tablet	Zero-order drug release kinetics
Hydrophobic (CW)	hydrophobic (carnauba wax)	Triple-layered tablet	Non-linear drug release
Hydrophobic (CW)	Hydrophilic (Methocel^®^ K15M) and Hydrophobic (CW)	Triple-layered tablet	Zero-order drug release kinetics.
Hydrophilic (HPMCAS&HPMC)	Hydrophobic (EC)	Triple-layered tablet	Zero-order release kinetics.

**Table 3:** Summary of various technologies that utilize geometric factors in drug delivery.

Technology	Design	Factors Affecting Drug Release	Type of Drug Release That May Be Achieved
Geomatrix^®^	Triple/bilayered tablet.	Type of polymer used, thickness of layers.	Zero-order kinetics
Smartrix^®^	Triple-layered tablet with core layer having a specific shape different to that of the outer layers.	Shape of core layer.	According to shape of core, zero-order kinetics
Procise^®^	Uniformly dispersed drug core containing a hole.	Geometry of core	According to geometry of core, zero-order kinetics
Dome Matrix^®^/“Release modules assemblage”	Elementary module containing a concave base side and a convex base side. Various arrangements of modules to form different structures	Arrangement of modules, type of polymeric material used.	Various according to arrangement of modules e.g., Concave base attached to concave base.
Core-in-cup devices	Disc-shaped core compression coated on one surface and circumference to form a cup around it.	Type of polymer, dimensions of core and cup.	Zero-order kinetics
Doughnut-shaped tablets	Single/triple-layered tablets with a central hole/holes	Size and number of holes, type of polymer used Zero-order kinetics Sodas^®^ Multilayer tablet Type of polymer used, thickness of layers. Shape of core layer	Pursatile drug release
VersaTab^®^	Bilayered tablet	Core drug, polymer layers	Immediate release and controlled release
Geolock^TM^	Triple layered tablet	Polymer layers, single or combination of drugs in the inner core	Immediate or modified release
